# General practice undergraduate and vocational training: ambulatory teaching and trainers’ curriculum and remuneration – a cross-sectional study among 30 member countries of WONCA Europe

**DOI:** 10.1186/s12909-023-04419-6

**Published:** 2023-06-14

**Authors:** Louise Devillers, Sébastien Friesse, Mette Caranta, Vincent Tarazona, Bastien Bourrion, Olivier Saint-Lary

**Affiliations:** 1 Department of General Medicine, Simone Veil University of Versailles Saint-Quentin-en- Yvelines and Paris-Saclay, Versailles, France; 2 Primary Care and Prevention Team, CESP, University Paris-Saclay, UVSQ, INSERM U1018, University Paris-Saclay, Villejuif, France

**Keywords:** Ambulatory teaching, General practice, Family medicine, Vocational training, Primary health care, Medical education

## Abstract

**Background:**

After a long phase without any propositions for real ambulatory training inside general practitioners’ offices, general practice (GP) vocational training has begun to appear progressively and has been integrated into undergraduate medical programmes. The aim of this study was to provide an overview of GP vocational training and GP trainers in member countries of the World Organization of National Colleges, Academies and Academic Associations of General Practitioners/Family Physicians (WONCA) Europe.

**Method:**

We carried out this cross-sectional study between September 2018 and March 2020. The participants responded to a questionnaire in real-life conversations, video conferences or e-mail exchanges. The respondents included GP trainers, teachers and general practitioners involved in the GP curriculum recruited during European GP congresses.

**Results:**

Representatives from 30 out of 45 WONCA Europe member countries responded to the questionnaire. Based on their responses, there is a well-established period for GP internships in undergraduate medical programmes, but with varying lengths. The programmes for some countries offer an internship after students graduate from medical school but before GP specialisation to ensure the career choice of the trainees. After specialisation, private practice GP internships are offered; however, in-hospital GP internships are more common. GP trainees no longer have a passive role during their internships. GP trainers are selected based on specific criteria and in countries, they have to follow some teacher training programmes. In addition to income from medical appointments carried out by GP trainees, GP trainers from some countries receive additional remuneration from various organisations.

**Conclusion:**

This study collected information on how undergraduate and postgraduate medical students are exposed to GP, how GP training is organised and the actual status of GP trainers among WONCA Europe member countries. Our exploration of GP training provides an update of the data collected by Isabel Santos and Vitor Ramos in the 1990s and describes some specificities that can inspire other organisations to prepare young, highly qualified general practitioners.

**Supplementary Information:**

The online version contains supplementary material available at 10.1186/s12909-023-04419-6.

## Introduction

Primary care, including general practice (GP), has evolved over the last 30 years while facing an increasing number of challenges. Its crucial role has been underlined by the World Health Organization (WHO) [1]. Meanwhile, universities have the responsibility of preparing tomorrow’s general practitioners. Universities are places of innovation, thanks in part to junior lecturers, who have contributed to an increasing number of publications in peer-reviewed journals, developing GP as an academic discipline [2]. In France, GP and family medicine are generally synonymous as disciplines. For clarity, in this article we use the terms GP and general practitioners. This choice is consistent with the World Organization of National Colleges, Academies and Academic Associations of General Practitioners/Family Physicians (WONCA) Europe definition [3].

The study conducted by Isabel Santos and Vitor Ramos in 1994 provided a relevant overview of GP training in 21 European countries [4]. This work might have been the impetus for several documents edited by WONCA Europe, its European Academy of Teachers in General Practice (EURACT) network [5] and the European Union of General Practitioners/Family Physicians (UEMO) [6], whose objective was to harmonise the process of becoming a GP trainer and receiving GP training during medical school. After a long phase without any proposals for real ambulatory training within general practitioners’ offices, GP training has started to appear progressively, contributing to the acknowledgment of GP as both a speciality and an academic discipline. While GP training was once only available for students who had graduated from medical school and had chosen the GP path, it has now become integrated into the curricula of undergraduate medical programmes [6].

Moreover, the organisation of GP training is still determined by the European Union (EU) Directives from 1993 [7] and 2005 [8]. To provide a high-quality education for future general practitioners, skilled GP trainers are necessary insofar as they develop and promote the most opportunities for trainees to be supervised in accredited practices.

Extending and upgrading French postgraduate programmes has recently been questioned. In fact, after a change in undergraduate medical programmes, students can now choose a second session for an internship in GP (like in other specialties) in their last year to increase their chances of obtaining the GP specialisation, which could be extended by one year.

The aim of this study was to provide an overview of GP vocational training and GP trainers of WONCA Europe member countries.

## Method

In this cross-sectional study, carried out between September 2018 and March 2020, we attempted to gather information on the organisation of GP vocational training and GP trainers of WONCA Europe member countries. We recruited general practitioners, GP trainers and teachers involved in the GP curriculum in Leuven, Belgium, in 2018 during the second EURACT Medical Education Conference, and in Tours and Nantes during the Collège National des Généralistes Enseignants (CNGE) Congresses in 2018 and 2019. We also contacted EURACT’s board members by email and asked them to participate. Finally, we recruited some respondents by the snowball effect. For this study, we contacted representatives of the 45 WONCA Europe member countries.

Two researchers conducted the individual interviews. The mode of data collection depended on the preferences of the respondents, using the same questionnaire: real-life conversations, video conferences (Skype®, Facetime® or WhatsApp®) and e-mail exchanges (Table 1). The researchers recorded all interviews, and the interviews were subsequently transcribed in their totality. When a respondent’s response required clarification, the researchers contacted them by e-mail. The researchers interviewed one respondent per country.

**Table 1:** Data collection method for each participating member country of the World Organization of National Colleges, Academies and Academic Associations of General Practitioners/Family Physicians Europe

Member country	Data collection method
Austria	E-mail exchange
Belgium	E-mail exchange
Bosnia and Herzegovina	E-mail exchange
Bulgaria	E-mail exchange
Croatia	26.55-minute phone call
Denmark	E-mail Exchange
Estonia	E-mail Exchange
Finland	E-mail Exchange
France	E-mail Exchange
Germany	21.32-minute phone call with follow-up e-mail exchange
Greece	E-mail exchange
Iceland	21.06-minute phone call with follow-up e-mail exchange
Israel	E-mail exchange
Italy	35-minute real-life conversation
Lithuania	E-mail exchange
Luxemburg	30-minute phone call
The Netherlands	20.35-minute video conference
North Macedonia	E-mail exchange
Norway	18.17-minute phone call
Poland	E-mail exchange
Portugal	E-mail exchange
Romania	E-mail exchange
Russia	E-mail exchange
Slovenia	21.01-minute phone call
Spain	E-mail exchange
Sweden	15.30-minute phone call
Switzerland	25-minute phone call
Turkey	E-mail exchange
Ukraine	26.40-minute phone call with follow-up e-mail exchange
United Kingdom	E-mail exchange

The questionnaire used for the interviews was based on the work of Dr I. Santos [4]. We translated the English version of the questionnaire into French and Danish. A PhD university lecturer provided proofreading and linguistic revisions and then cross-checked our French version of the questionnaire with the English version. We also used a Danish version because Danish is the native language of one of the researchers. The interviews included both closed and open questions on the following topics: general undergraduate programmes (medical school), GP postgraduate programmes and internships, formal status of the GP trainer, selection of trainers, training programmes, evaluation, assessment and remuneration for GP trainers (Supplementary File 1). In this work we defined postgraduate student as students who have graduated from medical school and has chosen a specialty.

We conducted a descriptive analysis of the interviews and compiled our results in tables. We did not perform statistical analysis because we did not attempt to compare answers among the countries.

## Results

**Fig. 1:**
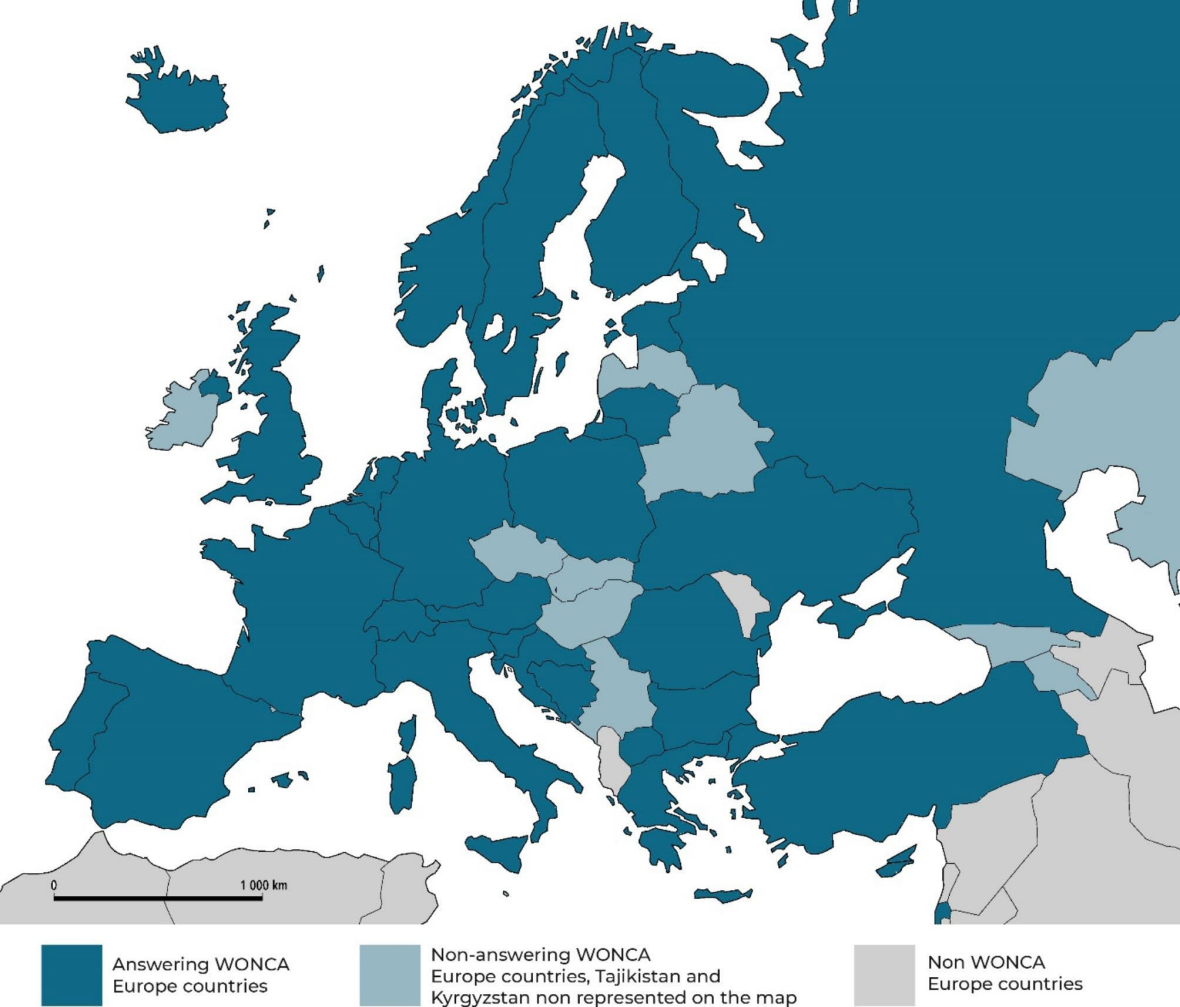
A map showing the World Organization of National Colleges, Academies and Academic Associations of General Practitioners/Family Physicians (WONCA) Europe member countries that responded to the questionnaire

Representatives of 30 out of 45 WONCA Europe member countries responded to the questionnaire (Fig. 1).

### GP training

#### Discovering GP in medical school

Based on the responses, the GP internships offered during undergraduate medical programmes are inconsistent (Table 2). The representatives of Ukraine, Russia, Italy and Greece reported that there are no undergraduate internships dedicated to GP. The Portuguese and Swiss respondents declared that although an internship is offered to undergraduate medical students, it is not mandatory. An internship could last from a few days (75 h in Bosnia and Herzegovina or 10 days in Denmark) to 4 months (the Netherlands). It should be noted that in Luxemburg, the undergraduate medical programme takes place at Luxemburg University for only the first two years, the GP internship during the following years relies on the university where the undergraduate medical student is enrolled: in France, Belgium or Germany.

**Table 2:** General practice internships in undergraduate medical programmes

Country	Undergraduate medical programme length (in years)	GP internship length (in months)
Austria	6	1
Belgium	6	1
Bosnia and Herzegovina	6	< 1
Bulgaria	6	Yes
Croatia	6	Yes
Denmark	6	< 1
Estonia	6	2
Finland	6	1
France	6	3
Germany	6	1.5
Greece	6	No
Iceland	6	1
Israel	6	1
Italy	6	No
Lithuania	6	Yes
Luxemburg	6	NA
The Netherlands	6	4
North Macedonia	6	Yes
Norway	6	1.5–2
Poland	6	2
Portugal	6	Yes
Romania	6	Yes
Russia	6	No
Slovenia	6	2
Spain	6	Yes
Sweden	5.5	1.5
Switzerland	6	Yes
Turkey	6	1.5
Ukraine	6	No
United Kingdom	5	Yes

**NA**, not applicable; **No**, no general practice internship declared; **Yes**, there is a general practice internship, but we did not get information on the length.

*GP internships for students who have graduated from medical school but not yet chosen a specialty: a rare opportunity*.

The representatives of 8 out of the 30 WONCA Europe member countries indicated that there are GP internships for students who have graduated from medical school but have not yet started specialisation (Table 3). The time dedicated to initiating a specialty ranges from 6 months (Slovenia) to 2 years (Finland). Students could participate in GP internships from 1 month (Austria) to 6 months (Finland). Slovenia does not offer a GP internship. In Denmark, Iceland and the Netherlands, students who have graduated from medical school have the opportunity to work as non-specialised doctors in designated places. After gaining this particular experience in the field, they can choose their specialty more wisely.

**Table 3:** General practice (GP) internships after graduating from medical school but before specialisation

Country	Length (in months)	GP internship length (in months)
Austria	8	1
Denmark	12	6
Finland	24	9
Iceland	12	4
Norway	18	6
Poland	12	2
Slovenia	6	0
Sweden	18–21	6

### GP as a speciality and internships in practices: GP vocational training

Among the WONCA Europe member countries, students who have graduated from medical school choose their specialty depending on their results and ranking after taking their exams and the availability of specialty training. The postgraduate GP training offered varies widely among the WONCA Europe member countries (Table 4).

**Table 4:** General practice (GP) postgraduate training

Country	Length (years)	GP internship (years)	Supervision of GP trainees by the GP trainer	Evaluation tools of GP trainees and pedagogical/professional resources for their specialisation
Austria	4	0.5	Indirect	Consultation logbooks and written exams
Belgium	3	2.5–3	Direct and indirect	Written exams and internship evaluation
Bosnia and Herzegovina	4	1.5–2	Direct	Written exams, interviews, scientific articles and reviews
Bulgaria	3	1.5	Direct	Skills certification, written exams, portfolios and internship reports
Croatia	4	2	Direct and indirect	Consultation logbooks, portfolios, skills certification and exams
Denmark	5	2.5 + 1 day per week during hospital internships	Direct, indirect and video	Skills certification and portfolio
Estonia	4	2	Direct	Consultation logbooks, exams and internship evaluations
Finland	6	2–4	Direct and indirect	Portfolios, workshops, Balint groups, e-learning and clinical skills evaluation
France	3	1–2	Direct and indirect	Portfolios and skills certification
Germany	5	2–3	Indirect	Workshops and final exams
Greece	5	2	Direct and indirect	Skills certification, consultation logbooks, written exams and objective structured clinical examination (OSCE)
Iceland	5	3	Direct, indirect and video	Skills certification and workshops
Israel	4	1.5	Direct and indirect	Skills certification, portfolios and written exams
Italy	3	1	Direct	Internship evaluation, written exams and thesis
Lithuania	4	1.5	No supervision, observation only	Portfolios and written exams
Luxemburg	3	≥ 1	Direct and indirect	Written exams, OSCE and oral presentations
The Netherlands	3	2	Direct and indirect	Portfolios and skills certification
North Macedonia	3	1	Direct and indirect	Skills certification, consultation logbooks and written exams
Norway	5	4	Direct, indirect and video	Written report cases, workshops and portfolios
Poland	4	2	Direct and indirect	Written exams
Portugal	4	3.5	Direct	Written and oral exams, skills certification and portfolios
Romania	4	3	Direct	Skills certification and written exams
Russia	2	18.5 weeks	Direct and indirect	Skills certification and written exams
Slovenia	4	2	Direct and indirect	Portfolio, skills certification, written exams and internship evaluation
Spain	4	2	Direct and indirect	Skills certification, consultation logbooks and e-portfolios
Sweden	4	2.5	Direct and indirect	portfolios, workshops and Balint groups
Switzerland	5	1	Indirect	Internship evaluation and written exams
Turkey	3	1.5	Direct and indirect	Oral and written exams and consultation logbooks
Ukraine	2	1	Direct	Written exams
United Kingdom	3	1.5	Direct and indirect	E-portfolios

In several countries, internships take place in practices, with one GP trainee per GP trainer’s office. Three trainers could mentor one trainee during their internship (with the weekly schedule and assignments organised by the trainers and their trainee), an approach that is similar to France. In Belgium, a GP trainee can participate in 5–6 GP internships, which represent the totality of the postgraduate programme. Only one 6-month-long non-mandatory internship can be carried out at the hospital. Internships in practices can also be extended for GP trainees in Portugal (three internships lasting 11, 22 and 11 months) and Israel (15 months during the first two years and the entire last year of the postgraduate curriculum). In Switzerland, the respondent stated that internships in practices tutored by ambulatory general practitioners are rare: only a few of them are GP trainers. They can last up to 1 year and are not mandatory. Internships take place in hospital units related to GP – in most cases, internal medicine departments. It is important to note that in Austria, GP is not yet considered a specialty; nevertheless, the respondent mentioned a 4-year postgraduate programme with a 6-month GP internship.

GP trainers can observe their trainees during consultations with their patients at the beginning of their internship, but stand-alone consultations with trainees are mostly preferred. In Israel and Moldova, the trainers are not present at the same practice while mentoring a trainee.

The respondents highlighted the many ways that the trainees’ progression is evaluated during their postgraduate programme, including written exams, practical exams and follow-up of skills acquisition during the internship reported in portfolios (Table 4). The respondents also mentioned pedagogical/professional resources for the GP specialisation, including mandatory participation in Balint groups in Sweden and Finland, writing and publishing scientific articles in Bosnia and Herzegovina and participation in workshops in Germany.

### Overview of GP trainers

Table 5 presents details on the GP trainers among the WONCA Europe member countries. The respondents for 21 of the 30 countries described a formal status for GP trainers, specifically an official status with the institutions involved in GP training including a university affiliation, GP departments or specific organisational assessments (e.g. state, specific ministries, a state’s department of health or medical chambers).

**Table 5:** General practice trainers

	Formal status for GP trainers	Affiliation	Selection criteria	Teacher training programmes		Remuneration
				Initial training	Lifelong training	
Austria	X	University and medical chamber	Yes	Yes	No	No
Belgium	X	University and FM department	Depends on the university, practice experience and GP specialist	2 days	1 day/year	No
Bosnia and Herzegovina	X	University, FM department and health ministry	10 years of practice and master in primary care	Continuous medical education	No	Yes, around 20% of their usual salary, from the health ministry
Bulgaria	No	University	3 years of practice and health ministry certification	Yes	No	Yes, from the government
Croatia	X	FM department and government	Yes	2 days	No	Yes
Denmark	No	With the region	Yes	2 days	1/5–7 years	No
Estonia	X	University	5 years of practice and accreditation needed	Yes	Yes	Yes
Finland	X	Yes	Yes	Yes	Yes	Yes
France	X	University and FM department, medical chamber	2 years of practice and accreditation needed	Yes, mandatory	Yes	Yes
Germany	X	Depends on the *länder* (state) and its competence centre	Depends on the *länder*	Depends on the *länder*	Depends on the *länder*	No
Greece	X	University and government	GP specialist and three publications	Yes	Yes	Yes, from the government
Iceland	No	No	Yes	Yes	1/5 years	Yes
Israel	X	Health maintenance organisation and FM department	Depends on the faculties	Yes, not mandatory	No	€250/month, from the health maintenance organisation
Italy	X	With the region	A few years of practice	Yes	No	Every month, from the government
Lithuania	X	University	Yes	No	No	Yes
Luxemburg	X	FM department	GP specialist and 5 years of practice	Yes	No	€200/month, from the government
The Netherlands	X	University	Evaluation of communications skills and knowledge	Yes, depends on the university	8 days/year	Yes + fees compensation
North Macedonia	No	No data	Yes	Yes	No	Yes
Norway	X	No	Evaluation by a state councillor	No	No	5% of their usual salary
Poland	X	University and health ministry	Yes	No	No	Yes + fees compensation
Portugal	X	No	2 years of practice	Yes, a short one	No	€520/month
Romania	No	No	Yes	Yes	No	No
Russia	No	No	Yes	No	No	No
Slovenia	X	University and medical chamber	Professional re-certification	2 days	Once a year	€180/month
Spain	X	FM department	Practice, teaching and research experience	Yes	Every year non mandatory	€200/month maximum, from health services
Sweden	No	With the region	Yes	Yes	2 days/year	No
Switzerland	No	University	Internist, GP or geriatrician for 3 years	1 day	4 h/2 years	From the university
Turkey	X	FM department	Exam results to shar and publications	Depends on the faculty	Depends on the faculty	From the ministry and university
Ukraine	No	No	No	No	No	No
United Kingdom	X	FM department	Completing the ‘certificate of medical education’	Yes	No	Annual fee/practice + pay Health Education England

For the ‘Formal status’ column, ‘X’ means that there is a formal status for GP trainers

For the ‘Affiliation’, ‘Selection criteria’, ‘Teacher training programmes’ and ‘Renumeration’ columns, ‘Yes’ means that it is relevant to the country, but we did not get specific details from the respondent

FM, family medicine

The criteria to become a GP trainer varies among the countries. The GP has to attest to anywhere from 2 years (France and Portugal) to 10 years (Bosnia and Herzegovina) of practice and obtain accreditation to host internships and GP trainees in their practice. In three countries, the research curriculum of the general practitioner is important: from a one-time research experience in Spain to the necessity to publish articles in Turkey and Greece (three publications needed). In the United Kingdom, a GP trainer must have a certificate in medical education. In the Netherlands, general practitioners have to pass a knowledge and communications skills test to become a GP trainer.

WONCA Europe member countries offer an initial training programme to prepare general practitioners for their new training role, except Lithuania, Norway, Poland, Russia and Ukraine. Wherever offered, lifelong training is implemented unequally, spanning from one session per semester in Sweden to 5 and 7 years in Denmark and Iceland, respectively. These training programmes are not mandatory for most countries and there are still a lot of countries where they are not organised.

In addition to the income trainees receive from their medical appointments, GP trainers receive remuneration from various sources; the amounts vary considerably from one country to another. In the Netherlands, this renumeration is for fee compensation or reinvestment of expenses. The representatives from Austria, Belgium, Denmark and Germany stated that the GP trainers have to contribute to the trainees’ salaries.

## Discussion

We collected information on how undergraduate and postgraduate medical students are exposed to GP, how GP training is organised and the actual status of GP trainers among WONCA Europe member countries. We offer a point-in-time view of the situation, and our findings are consistent with previous work involving some European countries [9]. Our exploration of GP training provides an update of the data collected by Isabel Santos and Vitor Ramos in the 1990s.

GP holds increasing importance in education programmes for medical students. Orienting medical students towards private practice might encourage careers in these fields [10]. More importantly, GP-related education provides future doctors with the opportunity to know the role of a general practitioner in health systems and primary care.

On the basis of a study regarding the efficiency of primary care – which involved most WONCA Europe member countries [11] – we understand that developing and improving GP training could play a role in reinforcing indicators like primary care governance and workforce development to improve the quality of primary care and general health for patients. It is important to focus on this second indicator, as studies have shown that GP training helps to motivate young doctors to become general practitioners [12] and to set up their practices in rural areas [13]. The better the GP experience across undergraduate medical programmes through curriculum and training development is, the stronger the will of students to build a career as a general practitioner [14].

Some good educational practices regarding the core values of GP have already been described in literature. A mix of educational tools is employed considering some essential principles such as the use of role models and contact with patients. With this experience, GP trainees can obtain the essential core values, knowledge, skills and attitude to become a general practitioner. This can be strengthened even more by allowing trainees and trainers to inspire each other and thus to share innovative educational methods [15]. Integration of GP education and training generally has a positive impact on all levels of learners [16].

Our results offer an overview of GP training and options to help shape a fourth year of GP specialisation in France while meeting the trainees’ educational needs. This approach could facilitate developing revalidation and reliable, high-quality guidelines [17] that produce wiser, qualified general practitioners willing to start their own practices. In France, a recent cohort study highlighted that training supervision is associated with an increase in the density of general practitioners in the municipality of practice. Training supervision seems to improve the attractiveness of a territory [18]. An increasing number of GP set-ups could increase the supply of primary care physicians and improve the continuity of care for patients. Indeed, it has been proved that a greater number of primary care physicians in a designated population is associated with reduced cardiovascular, cancer-induced and respiratory mortality [19]. Health care continuity provided by a general practitioner is associated with decreased mortality in general [20, 21].

Our study has several strengths. First, the questionnaire we used for the interviews is based on the work of Dr I. Santos (4): we translated the English version into French and Danish. There was a systematic and individual analysis and clarification of the answers given, which gave the authors the opportunity to confirm that the respondents had a good understanding of the questions. Second, collecting information from real-life conversations, video conference interviews and e-mail exchanges rather than a one-time online questionnaire without the ability to follow up gave us the opportunity to gather more precise information and sometimes details for which we did not ask. Our exchanges with the respondents provided a rich source of data that we could analyse.

There are some limitations to our study. First, we did not manage to collect information from all WONCA Europe member countries, nor did we have the opportunity to question our contacts about why they did not respond to our inquiries. Second, the results of this overview might be a little different from each country’s legal description of a GP training organisation. This discrepancy could be explained by the respondents’ own perceptions and interpretations of their system, which might have been influenced by various factors but also explained by the fact that it can take time to apply legislation and new guidelines in a well-rooted system. Finally, we interviewed only one representative per country, which means that we lacked specific information from certain regions or universities. For example, selection criteria and GP training organisations could be different for each region and university in a country. Indeed, the respondents from Belgium, Germany, Italy and Sweden voluntarily mentioned those possible specificities, although they did not have all the details.

## Conclusion

Our study illuminates GP training proposals and organisations among the members of WONCA Europe. Our overall picture of the situation provides original information that could be adapted to more countries in order to enhance GP training programmes. The same work could be carried out in more countries, beyond the borders of WONCA Europe, opening our perspectives to different patterns of GP training and GP as an academic discipline.

## Electronic supplementary material

Below is the link to the electronic supplementary material.

Supplementary Material 1

## Data Availability

The authors agree to provide full anonymised interviews if requested by the journal or a reader. Please contact the corresponding author: Dr Louise Devillers (louise.devillers@universite-paris-saclay.fr).

## Abbreviations

CNGE: Collège National des Généralistes Enseignants
EU: European Union
EURACT: European Academy of Teachers in General Practice
GP: General practice
NA: Not applicable
UEMO: European Union of General Practitioners/Family Physicians
UK: United Kingdom
WHO: World Health Organization
WONCA: World Organization of National Colleges, Academies and Academic Associations of General Practitioners/Family Physicians
