# Supplementary material for: General practice undergraduate and vocational training: ambulatory teaching and trainers’ curriculum and remuneration – a cross-sectional study among 30 member countries of WONCA Europe
Source: BMC Med Educ. 2023 Jun 14;23:439. doi: 10.1186/s12909-023-04419-6 (PMC10268461; doi:10.1186/s12909-023-04419-6)
Supplement: Supplementary file 1 — Supplementary Material 1 [file 12909_2023_4419_MOESM1_ESM.docx]

Questionnaire GP training:

Name:

Country/region:

City:

Status:

***First, I would like to ask you some questions about the general medical education system in your country***:

General Undergraduate program (basic medical education) in your country:

o How long does the undergraduate program last in your country?

o Are there clinical training in the General Practice office for undergraduates?

o How do undergraduates choose their specialty and residency program after graduation?

GP-postgraduate programme in your country:

o Is family medicine recognised as a specialty in your country?

o What is the duration of family medicine speciality training in your country?

o What is the duration of the rotation in GP practice?

o Does the residents choose their trainer or are this distribution organized by the faculty of medicine, minister of health etc?

o Is their supervision of the trainee? If yes, is it direct or indirect supervision?

o How are GP residents assessed? (Assessment of competencies, logbook, learning portfolio, formal exams, formative feedback, etc.?)

***Now, some questions on the organisation of the GP trainer in your country:***

Formal status of the GP trainer:

o Is there a formal status of the GP trainer?

o Is the GP trainer attached to a faculty of medicine or a GP department?

o Is there a written contract with the GP department of the medical school or with the government?

Selection of trainers:

o How does the selection of the trainers occur?

o Can anyone apply to become a trainer or are there criteria for the selection of trainers and practices for specific GP training?

Training programmes for trainers:

o Is there a mandatory teacher-training programme in your country (day-courses, workshops, etc.)?

Evaluation of trainers:

o Is there mandatory continuous assessment of the educational performance of trainers?

Remuneration:

o How much and how are GP trainers remunerated? Where does the funding come from? (Government, social security organisation, university, health care institutions, medical association, etc.?)

***Are there some specificities of GP training in your country that you would like to add?***

***Finally, do you know if there is an official document or website in your country that displays all this information on this subject?***

Thank you very much for your time.
